# 82125 Multiple epidemics of multidrug-resistant tuberculosis revealed by spatial disease mapping and whole-genome sequencing analysis in urban China

**DOI:** 10.1017/cts.2021.416

**Published:** 2021-03-30

**Authors:** Chongguang Yang, Yangyi Zhang, Yuan Jiang, Zheyuan Wu, Xin Shen, Joshua L. Warren, Ted Cohen

**Affiliations:** 1 Yale University School of Public Health; 2 Shanghai Municipal Center for Disease Control and Prevention

## Abstract

ABSTRACT IMPACT: Our study will integrate state-of-the-art methods in pathogen genomics, epidemiology, and geospatial analysis to identify both host- and pathogen-factors driving the MDR-TB transmission and the study outcome can inform the design of targeted interventions OBJECTIVES/GOALS: The emergence of multidrug-resistant tuberculosis (MDR-TB) poses serious challenges for the global eradication of tuberculosis. Recent research has shown that transmission is now the dominant driver of MDR-TB. However, our limited understanding of where and among whom MDR-TB is transmitted hampers efforts to control person-to-person spread. METHODS/STUDY POPULATION: We used several analytic approaches to characterize the dynamics of MDR-TB transmission in Shanghai, China. We identified all culture-confirmed MDR cases between 2009-2016 in the city and 1) estimated individual-level risk factors for MDR disease; 2) mapped the TB cases by their home addresses and used a Bayesian spatial disease mapping method to identify regions with an elevated risk of MDR-TB; and 3) we sequenced all MDR isolates to understand whether transmission explained variance in risk that was not attributable to the distribution of individual or location-specific risk variates. RESULTS/ANTICIPATED RESULTS: There were 1034 MDR-TB cases among 16,315 culture-confirmed TB cases during the study period. Bayesian disease mapping identified spatial heterogeneity of MDR-TB and determined four hotspots with an elevated risk of MDR-TB, none of which were fully explained by individual or regional-covariates (Figure 1). Sequencing revealed that more than 40% of the MDR-TB strains were in genomic clusters, indicating recent MDR-TB transmission. Most importantly, MDR-TB cases in three of the four large clades (>8 isolates) were spatially concentrated in three strain-specific hotspots (Figure 2). DISCUSSION/SIGNIFICANCE OF FINDINGS: With the combination of traditional epidemiological tools, geographical, and genomic methods, this study revealed multiple loci of transmission of specific MDR-TB clades within a single city. Identification of where and among whom MDR-TB is transmitted can inform the design of targeted interventions.

